# Organic versus functional dichotomy in gastroenterology: Are we ready to move the needle?

**DOI:** 10.1002/ueg2.12588

**Published:** 2024-05-17

**Authors:** Daniel Keszthelyi

**Affiliations:** ^1^ Department of Gastroenterology‐Hepatology Maastricht University Medical Center+ Maastricht the Netherlands

**Keywords:** disorders of the gut‐brain interaction, functional dyspepsia, functional gastrointestinal disorders, irritable bowel syndrome

According to the French philosopher René Descartes (1596–1650), mind and body are completely different from one another, and each can exist by itself. This shift in thought was congruent with the societal changes of the 17th century: the separation of church and state. With this, Descartes demythologized the human body to circumnavigate religious boundaries and paved the way for progress in medical science through the study of physiology and anatomy of the human body that gave birth to modern medicine. Unfortunately, by ignoring the mind, the strong divergence of mental health care from more conventional medicine became a byproduct. Later on, when psychoanalysis took over psychiatry in Europe and in the USA during the early 20th century, psychiatry has gradually become separated from the rest of the medical specialties.^1^

Traditionally, diseases that are assumed to be based on structural bodily abnormalities are termed “organic,” and in their apparent absence, the term “functional” is used, reflecting the Cartesian dichotomy. Admittedly, no biomarker for irritable bowel syndrome (IBS) and similar functional disorders has been identified, and diagnosis is therefore symptom‐based to this day. At the same time, the term “functional” became strongly associated with psychiatry, oftentimes being used as well‐intended yet stigmatizing euphemism for psychiatric comorbidities.

Cartesian dualism has been subject to criticism ever since its inception. Neither did it go unchallenged in that time. Princess Elizabeth of Bohemia (1618–1680) in her correspondence with Descartes raised the questions “given that the soul of a human being is only a thinking substance, how can it affect bodily spirits, in order to bring about voluntary actions^2^?” In another words: for the mind to affect the body, wouldn't it need to make contact with the body? Elizabeth never received a satisfactory answer from Descartes.

Several attempts have since been made to resolve body‐mind dualism, most importantly the bio‐psycho‐social model introduced by George Engel in 1977.^3^ The bio‐psycho‐social model has also served as inspiration for the re‐conceptualization of “functional” gastrointestinal (GI) disorders. Accordingly, in order to minimize the stigmatizing effects of this nosology, the Rome Foundation has in its last iteration of the Rome criteria in 2016 introduced the term “disorders of the gut‐brain interaction (DGBI), also reflecting the evolving understanding of the pathological processes underlying symptom generation in these disorders.^4^”

The applicability of the term DGBI, however, has recently been questioned.^5^ Conditions such as hepatic encephalopathy, traditionally not considered a functional GI disorder, better seem to conceptually fit within the framework of DGBI than the “traditional” functional GI disorders, such as IBS.

Neither does the term DGBI fully reconcile the large overlap between “organic” and “functional” GI disorders. Indeed, in this issue of the journal, van Gils et al.,^6^ using data from the Rome Foundation Global Epidemiological Study (an internet survey performed in 26 countries including over 54 thousand subjects), showed strong associations between self‐reported history of an organic disease (including reflux disease, celiac disease, inflammatory bowel disease and diabetes mellitus) and DGBI‐compatible symptom profiles. The authors found that those individuals with a history of organic disease and at least one DGBI‐compatible symptom profile had higher level of psychological distress and lower quality of life compared to those without any DGBI‐compatible profile. However, these differences were not specific to individuals with organic diseases, since the same patterns was seen in the overall survey sample. These findings are in line with recent reports in the field of reflux disease, showing that psychological symptoms do not significantly discriminate between reflux phenotypes, regardless of “organic” versus “functional” nature of the symptoms.^7^

The fact that symptom profiles are unable to distinguish organic and functional disorders from each other adds to the notion that such distinction is not particularly helpful in clinical practice either. So rather than merely warping functional GI disorders into a more appropriate term, our efforts should also be directed at accepting the fact that all patients have unique symptom experiences impacting their general and mental well‐being, regardless whether a specific “organic” origin has been identified for these symptoms. Perhaps once scientific progress identifies the different processes underlying symptom generation, for instance related to the disturbances in body‐brain interactions, such as recently discovered,^8^ and we are able to detect these alterations as a part of routine clinical diagnostic work‐up, the term “functional” will only find its relevance in history books.

## CONFLICT OF INTEREST STATEMENT

The authors have no conflicts of interest to declare.

## Data Availability

Data sharing is not applicable to this article as no new data were created or analyzed in this study.
